# Working Memory for Linguistic and Non-linguistic Manual Gestures: Evidence, Theory, and Application

**DOI:** 10.3389/fpsyg.2018.00679

**Published:** 2018-05-15

**Authors:** Mary Rudner

**Affiliations:** Linnaeus Centre HEAD, Swedish Institute for Disability Research, Department of Behavioural Sciences and Learning, Linköping University, Linköping, Sweden

**Keywords:** working memory, manual gestures, sign language, deafness, semantics, phonology, cochlear implantation

## Abstract

Linguistic manual gestures are the basis of sign languages used by deaf individuals. Working memory and language processing are intimately connected and thus when language is gesture-based, it is important to understand related working memory mechanisms. This article reviews work on working memory for linguistic and non-linguistic manual gestures and discusses theoretical and applied implications. Empirical evidence shows that there are effects of load and stimulus degradation on working memory for manual gestures. These effects are similar to those found for working memory for speech-based language. Further, there are effects of pre-existing linguistic representation that are partially similar across language modalities. But above all, deaf signers score higher than hearing non-signers on an n-back task with sign-based stimuli, irrespective of their semantic and phonological content, but not with non-linguistic manual actions. This pattern may be partially explained by recent findings relating to cross-modal plasticity in deaf individuals. It suggests that in linguistic gesture-based working memory, semantic aspects may outweigh phonological aspects when processing takes place under challenging conditions. The close association between working memory and language development should be taken into account in understanding and alleviating the challenges faced by deaf children growing up with cochlear implants as well as other clinical populations.

## Working Memory and Language

Working memory is the ability to simultaneously store and process information (Daneman and Carpenter, 1980; Baddeley, 2000; Ma et al., 2014) and as such forms the foundation of higher cognition including thinking and learning. Working memory provides a platform for language processing by keeping information in mind and integrating it with new information during discourse processing as well as a platform for language learning (Baddeley et al., 1998), i.e., the establishment of new linguistic representations. The storage and processing limits of working memory may constrain language processing when it takes place under challenging conditions, i.e., when the incoming language signal is degraded and therefore cannot be readily matched to existing representations (Rönnberg, 2003; Rönnberg et al., 2008, 2013). For most people, language is primarily speech-based. However, for individuals with reduced hearing ability, gesture-based language, i.e., sign languages, provide an alternative means of communication that bypasses the defective auditory channel. The communicative importance of sign language gives the study of working memory for manual gestures applied significance. However, it also has a theoretical interest as gestural and vocal communication seem to share common origins (Corballis, 2003; Aboitiz and García, 2009; Aboitiz, 2012) and their comparison can provide insight into the architecture of working memory and its language modality specificity.

### Working Memory for Sign and Speech

The comparison of working memory for sign language to working memory for speech has demonstrated similar capacity across language modalities (Boutla et al., 2004; Andin et al., 2013) and similar lifespan trajectories (Rudner et al., 2010). However, when processing demands are low and maintenance demands high, sign capacity is more similar to visuospatial capacity (5+/-2) than speech-based verbal capacity (7+/-2, Boutla et al., 2004; Andin et al., 2013). There are also language-modality specific differences in the neural networks supporting working memory for sign and speech. Specifically, working memory for sign generates more activation compared to working memory for speech in superior parietal regions associated with visuospatial processing and temporo-occipital regions associated with object recognition (for a review see Rudner et al., 2009). This net activation for sign language may reflect sign specific sensorimotor mechanisms and modes of representation (Emmorey et al., 2014) or language modality-specific executive strategies employed during working-memory tasks (Bavelier et al., 2008). Specifically, net superior parietal activation may reflect generation and storage of a virtual spatial array (Rönnberg et al., 2004, 2008; Rudner et al., 2009) in, or close to, a neural region identified as a capacity-limited store for representation of the visual scene (Todd and Marois, 2004, see Rudner, 2015 for discussion). Further, a recent animal study has shown that visuospatial working memory in deaf individuals is dependent on parietal cortex (Wong et al., 2017). Rather than focusing on the comparison of working memory for sign to working memory for speech, the current review targets work investigating working memory for manual gestures that may or may not be familiar and/or lexicalized in a sign language.

### Linguistic and Non-linguistic Manual Gestures

In sign languages, use of gestures is formalized in lexicon and grammar but also in manual alphabets. The latter represent the orthography of written languages and are used in the context of sign languages for finger-spelling names or concepts that have not acquired their own sign. Deaf individuals who lack formal language input often use conventionalized gestures to communicate. These are known as homesigns (Spaepen et al., 2013). Manual gestures can function as emblems (Ekman and Friesen, 1969), e.g., “thumbs up” or provide emphasis in the context of spoken language (Chieffi and Ricci, 2005). They facilitate discourse comprehension (Wu and Coulson, 2014) and production (Morsella and Krauss, 2004; Gillespie et al., 2014) by relieving pressure on challenges to working memory (Wesp et al., 2001; Morsella and Krauss, 2004; Ping and Goldin-Meadow, 2010; Chu et al., 2014), especially for individuals with low working memory capacity (Marstaller and Burianová, 2013). Simple manual actions, however, may be used without semantic intent. Importantly, manual actions can be encoded, stored and rehearsed in working memory, irrespective of whether they have the status of manual gestures or signs (Wilson and Fox, 2007; Rudner, 2015). However, they do not seem to have the same role as gestures in supporting speech processing (Cook et al., 2012). Notwithstanding, this means that by carefully selecting manual actions, gestures and signs, linguistic working memory in the visual domain can be systematically investigated.

### Semantics and Phonology

All languages have a lexicon and sign languages have lexicons that consist of signs constituted by manual actions that by definition are associated with meaning. Furthermore, within any given language-specific lexicon, individual items are consistent with the phonology of that language: this also applies to sign languages. Whereas in speech-based languages phonology can be defined either in terms of the patterning of sounds adopted within that language, or the articulatory gestures involved in producing those sounds, in signed languages an articulatory definition is adopted. Sign language phonology is defined as the patterning of formational aspects of individual signs including shape, movement and location in relation to the body (Stokoe, 1960; Sutton-Spence and Woll, 1999). In other words, handshape, movement and location are all contrastive elements that constitute the sublexical components of lexical signs. This means that any lexical sign not only has a specific meaning (or set of meanings) but also a specific phonological composition that distinguishes it from all other signs in the lexicon. However, for a person who is not familiar with any sign language, a manual gesture may not signify anything at all, and further, its sublexical composition may not comply with any known set of rules. Thus, for the non-signer, any given sign may lack both semantics and phonology. This state of affairs makes manual gestures an excellent tool for investigating the effects of semantics and phonology on working memory.

## Working Memory for Manual Gestures

### Measuring Working Memory for Gestures

**FIGURE 1:**
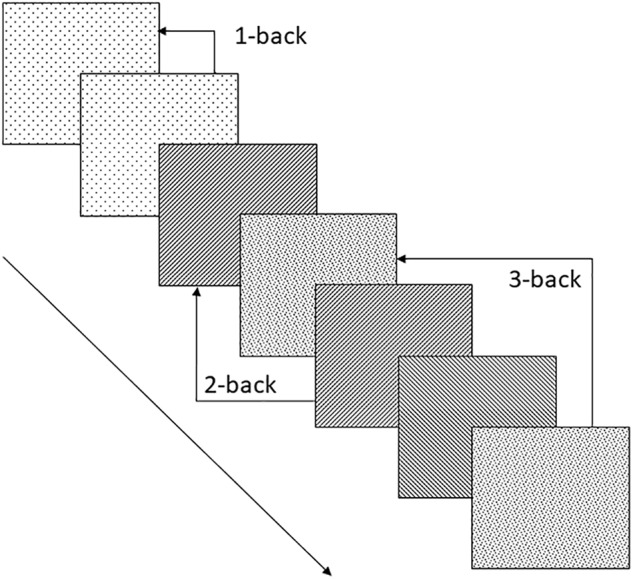
Schematic representation of the n-back working memory task with examples of 1-, 2- and 3-back matches. Each square represents a visual stimulus that may be for example a video-recorded sign or a picture of an object. The pattern in each square represents a given characteristic of the stimulus which may be the stimulus as a whole, a surface feature of the stimulus such sign handshape (if the stimulus is a video-recorded sign) or an inferred feature of the stimulus such as the handshape of the sign gloss of a depicted object.

Although non-signers can encode manual gestures in memory (Wilson and Fox, 2007; Rudner, 2015), they are less successful than signers at imitating them (Holmer et al., 2016b) and thus at a disadvantage when it comes to recall of encoded signs (Rudner, 2015; Rudner et al., 2016b). The n-back paradigm (Cohen et al., 1994; Owen et al., 2005) avoids the confounding effects of suboptimal imitation ability on the production demands of traditional serial recall paradigms, and can be used to investigate working memory for both linguistic and non-linguistic manual gestures (for examples see **Table 1**). In the n-back paradigm, memoranda are presented serially and the participant is asked to match each item as it occurs to the stimulus that was presented n steps back in the series, see **Figure 1**. Typically, *n* = 2 is considered to generate moderate working memory load (Smith and Jonides, 1997), requiring the maintenance of two items and their order along with the simultaneous processing demands of matching each new stimulus to the first item maintained in the storage buffer, and then updating the buffer. Updating the buffer involves adding the new item as the second item in the buffer after the original second item moves up to first place and the now obsolete item that was formerly the first item in the storage buffer is suppressed or deleted. Working memory load can be manipulated in the n-back paradigm by adjusting n. *N* = 1 is considered a low memory load and *n* = 3 high memory load (Smith and Jonides, 1997).

**Table 1:** Overview of studies using the n-back paradigm to investigate working memory for linguistic and non-linguistic manual gestures.

Reference	Method	Stimulus material	Sample	Working memory Load (n)	Additional design factors	Results
Rudner, 2015	Behavioral	Video-recorded meaningless manual gestures	20 hearing non-signers	1, 2	Location (proximal, distal)	At low load, gesture proximity reduced performance
Rudner et al., 2015	Behavioral	Video-recorded lexical signs	20 hearing non-signers	1, 2	Resolution at 5 levels	Low resolution reduced performance more when load was high
Rudner et al., 2007	fMRI	Video-recorded lexical signs and words	13 Hearing native signers	2	Match (sign-sign, word-word, sign-word)	Cross modal processing, activated posterior regions including the right middle temporal lobe, possibly relating to binding of phonological loop representations with semantic representations in long-term memory
Rudner et al., 2013	fMRI	Pictures	11 deaf signers, 20 hearing non-signers	2	Semantic, phonological, orthograhic	Poorer behavioral performance on phonological and orthographic than semantic conditions. Distinct neural networks at all 3 levels of linguistic processing modality-specific differences
Rudner et al., 2016b	Behavioral	Video-recorded familiar and unfamiliar signs as well as non-signs and non-linguistic manual actions	24 deaf signers, 20 hearing signers, 24 hearing non-signers	1, 2, 3	Load and material manipulated orthogonally	Hearing signers performed better with familiar than non-familiar signs. Deaf signers also performed better with familiar than non-familiar signs but only when load was high. Deaf signers performed better than hearing non-signers with all materials except non-linguistic manual actions
Cardin et al., 2017	fMRI	Dynamic point-light displays of lexical signs or nonsense objects	12 deaf signers, 16 hearing native signers, 16 hearing non-signers	2	Attentional control task with both materials	Deaf signers showed more posterior temporal and less fronto-parietal activation as well as increased resting state connectivity between frontal and temporal regions. These effects were independent of the linguistic characteristics of the stimuli

### Pre-existing Semantic Representation Increases Working Memory Capacity Across the Language Modalities of Sign and Speech

**FIGURE 2:**
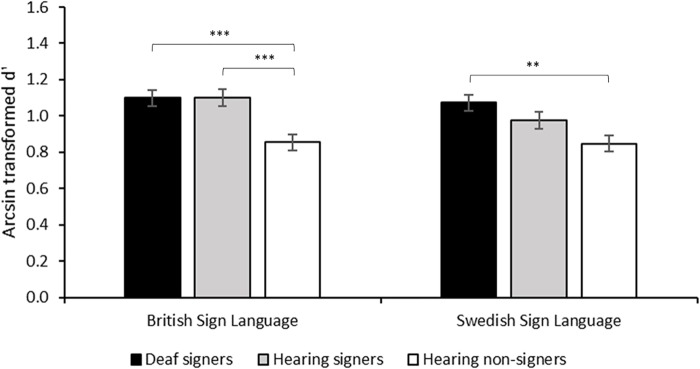
Working memory performance with British Sign Language (BSL) and Swedish Sign Language (SSL) stimuli by British Deaf Signers (DS), Hearing British Signers (HS) and Hearing British non-signers. The *y*-axis shows arcsin transformed d′ collapsed across load. D′ is based on hits adjusted for false alarms in accordance with signal detection theory, and the arcsin transformation was used because of near ceiling performance at *n* = 1. Reprinted with the publisher’s permission from Rudner et al. (2016b).

Working memory capacity for words is greater than capacity for pseudowords (Hulme et al., 1991). Recently, it has been shown that for British Sign Language (BSL) users this effect generalizes to lexical signs, demonstrating that the positive effect of pre-existing semantic representation on working memory capacity generalizes to sign language (Rudner et al., 2016b, see **Table 1**). In particular, BSL users, both deaf and hearing, scored higher than British non-signers on an n-back task based on video-recorded manual gestures (see **Figure 2**). More critically, hearing BSL signers scored better when items were lexicalized in BSL than when they were not. This applied across memory loads (*n* = 1–3) and irrespective of whether the non-British signs were lexicalized in another mutually unintelligible sign language, Swedish Sign Language (SSL), made-up non-signs or non-linguistic manual actions. A similar pattern was found for British deaf signers, although here the difference in performance on the n-back task between BSL and SSL was only significant when working memory load was high (*n* = 3).

### No Evidence That Pre-existing Phonological Representation Supports Working Memory for Manual Gestures

Not only pre-existing semantic representation but also pre-existing phonological representation enhances working memory for words (Gathercole et al., 1999). However, review of the literature reveals no evidence that this effect generalizes to sign language. Thus, the effect of pre-existing phonological representation on working memory for words appears to be language-modality specific.

The potential effect of pre-existing phonological representation on working memory for signs was investigated by Rudner et al. (2016b, see **Table 1**) in two different ways. In the first place, it was tested whether BSL signers had higher scores than non-signers on the n-back task when the stimuli had accessible phonology, and in the second place, whether the signers performed better when stimuli had accessible phonology than when they did not. Items with accessible phonology were SSL, that is, they were lexicalized in SSL and were thus real natural signs but not lexicalized in BSL and thus lacked meaning for the British signing participants.

The phonological repertoires of BSL and SSL are highly similar (Rudner et al., 2016b) and thus even though the semantics of the SSL items was not available to the signing participants, its phonological structure was. SSL was contrasted with artificially constructed phonologically illegal non-signs that were eligible for lexicalization in neither BSL nor SSL. The deaf signers who participated in the study did indeed score significantly better than the hearing non-signers on the n-back task with SSL, in line with our prediction of modality generality, but a similar (although non-predicted) performance discrepancy was also found with non-signs. This suggested that pre-existing phonological representation was not the true cause of the effect. Further, there was no difference in performance with either SSL or non-signs between the two hearing groups. Because hearing signers are just as likely to benefit from access to sign phonology as deaf signers it seems unlikely that the working memory advantage of the deaf signers over hearing non-signers with SSL was caused by pre-existing phonological representation.

A sharper test of the potential effect of pre-existing phonological representation on working memory for manual actions, however, was the within-group comparison of n-back scores for SSL and non-signs for the two signing groups. We aimed to isolate the effect of pre-existing phonological representation by comparing n-back scores with SSL, an unfamiliar but phonologically accessible sign language, with n-back scores for non-signs that were deliberately created to contravene the phonological constraints of BSL. However, the phonology of sign language carries semantic information in a way that the phonology of spoken language does not. For example, signs may be iconic, i.e., have visual similarity to the objects they represent, e.g., the sign for aeroplane depicts wings and upward movement (BSL example from Thompson et al., 2012). This means that the signs of even an unfamiliar sign language may carry semantic information. Although the SSL signs were selected to be semantically opaque to the British participants, it is possible that the phonological features of the SSL signs did provide some semantic cues that could be deployed mnemonically during the n-back task. This made for a conservative comparison between n-back scores with BSL and SSL, but at the same time, it made the comparison of SSL to non-signs rather liberal. Even so, there was no difference in performance between these two stimulus types for the signing groups and for the non-signing group, to whom iconic features would also be available, there was even a tendency toward an advantage of non-signs over SSL. Thus, Rudner et al. (2016b) found little support for the notion that pre-existing phonological representation supports working memory processing.

### Deaf Signers Have Greater Working Memory Capacity for Sign-Based Gestures Than Hearing Non-signers

Signers are experts in using their own language and thus it is hardly surprising that they show better performance than non-signers on a sign-based working memory task due to their expert knowledge of the language (Ericsson and Kintsch, 1995). As I have argued, part of that benefit derives from the pre-existence of semantic representations (Rudner et al., 2016b). However, above and beyond that benefit there seems to be an additional advantage for deaf signers that does not pertain specifically to pre-existing phonological representation and is not apparent for hearing signers. Deaf individuals are highly reliant on visual information for perception and communication. Therefore, it is not surprising that they develop special skills in the visual domain. Low level visual processing does not seem to be enhanced in deaf individuals but for visual skills with a greater cognitive component, such as visual attention, congenitally deaf individuals do show some advantage that is associated with neural plasticity (for a review see Bavelier et al., 2006 and discussion Rudner et al., 2009).

## Cross-Modal Plasticity

When sensory cortex is not recruited in its typical mode during development, cross-modal plasticity takes place (Merabet and Pascual-Leone, 2010). This applies to both visual cortex in the occipital lobe (Kupers and Ptito, 2014) and auditory cortex in the temporal lobe (Nishimura et al., 1999; Finney et al., 2001; Cardin et al., 2013) in humans. Deaf humans recruit right auditory cortex more than hearing individuals during observation of dynamic visual but not linguistic stimuli (Finney et al., 2001) and the superior temporal cortex bilaterally during observation of signs (Nishimura et al., 1999). Cardin et al. (2013) dissociated perceptual and cognitive effects, showing that while right superior temporal cortex reorganizes to process non-linguistic dynamic visual stimuli irrespective of linguistic content, the left superior temporal cortex is only sensitive to dynamic visual stimuli with linguistic content (Cardin et al., 2013). Animal studies have shown that the regional localization of cross-modally reorganized functions can be very specific: congenitally deaf cats have better orientation abilities in the visual periphery than hearing cats but this benefit is suspended by deactivating regions of the temporal lobe by localized cooling. In particular, deactivation of posterior auditory cortex selectively eliminated their superior visual localization abilities, whereas deactivation of the dorsal auditory cortex eliminated their superior visual motion detection (Lomber et al., 2010). It is likely that the localization of visually based linguistic and cognitive functions reorganized in the auditory cortex of congenitally deaf humans is just as specific, and that with the right techniques it will be possible to localize these functions.

### Cross-Modal Plasticity in Temporal Cortex Supports Working Memory

There is accumulating evidence that the superior temporal cortex is engaged in working memory processing in deaf signers in a manner that is not observed in hearing individuals (Cardin et al., 2017, see **Table 1**; Ding et al., 2015). In particular, British deaf signers showed activation of the bilateral posterior superior temporal cortex during a 2-back working memory task, irrespective of whether it was based on BSL signs or moving nonsense objects (Cardin et al., 2017). This extended the work of Ding et al. (2015) using individuals with early deafness but with diverse language experience, by demonstrating that recruitment of superior temporal cortex in congenitally deaf individuals still takes place when language skills are well established and is thus not simply caused by poorly established language skills (MacSweeney and Cardin, 2015). Further, in the study by Cardin et al. (2017), the deaf compared to hearing participants showed increased resting state connectivity between frontal regions and the superior temporal cortex, and this finding was replicated by Ding et al. (2016) with deaf Chinese participants. These findings show that congenital deafness leads to reorganization of working memory networks. This extends previous findings showing that differences in working memory networks for sign and speech are influenced not only by language modality but also auditory deprivation.

The absence of activation differences between linguistic and non-linguistic working memory in the study by Cardin et al. (2017) confirms the suggestion of Ding et al. (2015) that the functional significance of the reorganized networks is related to visuospatial working memory rather than working memory for sign language as such. Nonetheless, these findings mean that the superior temporal cortex of congenitally deaf individuals reorganizes not only for perceptual processing of visual stimuli but also for their cognitive processing, such as working memory. This perceptual and cognitive reorganization may be related to the performance advantage of deaf signers over hearing non-signers on visual working memory tasks (Wilson et al., 1997; Geraci et al., 2008; MacSweeney and Cardin, 2015; Rudner et al., 2016b; Cardin et al., 2017).

## The Role of Phonology in Working Memory for Manual Gestures

It also needs to be considered why pre-existing phonological representation does not give signers a working memory advantage, at least not in an n-back task (Rudner et al., 2016b). The phonological composition of to-be-remembered speech-based items influences processing (Baddeley, 2000). In particular, phonological similarity between items decreases working memory performance. This effect is well-attested for speech-based items and there is also evidence that phonological similarity among American Sign Language (ASL) signs decreases short-term memory performance (Wilson and Emmorey, 1997). However, there is to my knowledge no evidence of such an effect for BSL (for discussion see Andin et al., 2013). Thus, although there is evidence that sign-based phonological similarity influences working memory processing, this may not generalize across all sign languages, including BSL. Further, there is evidence that phonological information may be suppressed during the n-back task when it is not explicitly required for task solution (Sweet et al., 2008; Rudner et al., 2016b). Such an effect may be enhanced in sign language as it has been pointed out that phonological information may be heavier in signed than spoken language (Geraci et al., 2008; Gozzi et al., 2010; Marshall et al., 2011) and thus there may be more incentive to ignore it if it is task irrelevant, particularly if a semantic route to task solution is effective.

Although, there is apparently no evidence of an effect of pre-existing phonological representation on working memory for unfamiliar signs in deaf British signers, there is evidence of an effect of phonological representation on phoneme monitoring in this population (Cardin et al., 2016). Using video-recorded BSL, SSL and non-sign stimuli similar to those used in the working memory study by Cardin et al. (2016), Rudner et al. (2016b) showed greater bilateral activation of an acknowledged phonological processing region, namely the supramarginal gyrus, for lexical signs compared to non-signs in deaf signers, i.e., in participants with pre-existing phonological representations. Supramarginal gyrus activation for the signers did not differ with the phonological parameters that were targeted in the task (handshape and location) and was thus phonology specific rather than task specific. This means that it is unlikely that the absence of an effect of pre-existing phonological representation on n-back score was due to an inability to access the phonological information contained in the stimuli. Indeed, the ability to explicitly access phonological representations of sign language has been demonstrated across sign languages not only in phoneme monitoring tasks (Gutierrez et al., 2012; Grosvald et al., 2012), but also in phonological similarity judgment tasks (MacSweeney et al., 2008b; Andin et al., 2014; Holmer et al., 2016b) and for SSL in a working memory context (Rudner et al., 2013, see **Table 1**). Instead, the likely explanation is that when the lexical signs were maintained in working memory, the phonological information associated with them was suppressed because it was irrelevant to task solution (Sweet et al., 2008) and may have increased working memory load (Marshall et al., 2011).

Intriguingly, the study by Cardin et al. (2016) showed no difference in neural activation between BSL and SSL for any of the groups. In other words, there was no significant effect of pre-existing semantic representation on phoneme monitoring (c.f. Petitto et al., 2000; Grosvald et al., 2012). This indicates that the significant effect of pre-existing semantic representation on n-back performance (Rudner et al., 2016b) is likely reserved for the context of the working memory task in which semantic encoding, when possible, reduced task demands.

### Working Memory for Non-linguistic Manual Actions

Although the combination of deafness and sign language experience conferred a working memory advantage during processing of familiar and unfamiliar signs as well as non-signs, it did not generalize to an advantage in working memory processing of non-linguistic manual actions consisting of ball-catching events (Rudner et al., 2016b). These ball-catching events were generated by asking the model who recorded the signs and non-signs to catch a small ball that was thrown toward him. Critically, the manual actions that were generated in this manner were elicited in a bottom–up rather than top–down fashion. The purpose of this was to eliminate intentionality from the actions. Performance on the n-back task was poorer with non-linguistic manual actions than with any of the other stimulus types. However, there was a significant effect of working memory load between each level of n (Rudner et al., 2016b) and a separate study showed an effect of formational similarity on n-back performance (Rudner, 2015, see **Table 1**). One perceptual difference between the non-linguistic manual actions and the signs and non-signs was the reduced motoric diversity displayed by the model. In particular, the handshape used to catch the ball was similar in all instances and although the ball was thrown to difference segments of the space around the model, the movements he made to catch the ball were stereotypical even if they differed in trajectory. Thus, the poorer performance by all groups with non-linguistic manual actions compared to non-signs and signs may be due to too little motoric diversity to distinguish separate items (c.f. Sehyr et al., 2017). This notion is supported by the effect of formational similarity on n-back performance with non-linguistic manual actions in which the degree of motoric diversity significantly influenced performance (Rudner, 2015).

## Working Memory Load – Effect Across Materials and Groups

Working memory load is increased when more items are maintained for the same amount of processing. This is achieved using the n-back paradigm by increasing the magnitude of n. An effect of working memory load has been observed for all types of manual gestures under consideration here: familiar signs, unfamiliar sign, non-signs and non-linguistic manual actions (Rudner, 2015; Rudner et al., 2015, 2016b). Interactions with load can be informative of the way in which different types of information are stored in working memory. In particular, a significant interaction between load and gesture type for deaf signers showed that for this group, the effect of pre-existing semantic representation was only apparent when working memory load was high (Rudner et al., 2016b). This was in contrast to hearing signers who showed an effect of pre-existing semantic representation across memory loads (Rudner et al., 2016b). Further, in the same study, another significant interaction between load and gesture type showed that although there was an effect of load for non-linguistic manual actions, in line with previous work (Rudner, 2015), it was lower than for non-signs (Rudner et al., 2016b).

## Speech-Based Recoding of Familiar Signs by Bimodal Bilinguals

The difference in the effect of semantic representation across memory loads between deaf and hearing signers (Rudner et al., 2016b) suggested that there were differences in working memory processing across to the two signing groups. There is evidence that working memory encoding and maintenance are more efficient for words than signs for bimodal bilinguals (Hall and Bavelier, 2011). Thus, it is likely that to maximize task performance the hearing signers in the study by Rudner et al. (2016b) encoded and maintained the familiar signs as words. On the other hand, the deaf signers who did not have such ready access to the speech modality most likely encoded and maintained the lexical signs in the visual language modality in which they were presented. Recently, it has been shown that deaf signers with good reading skills recode fingerspelled words as speech-based phonology during a working memory task (Sehyr et al., 2017). It is likely that the representations resulting from recoding by deaf signers are more fragile and susceptible to working memory load than those of the bimodal bilinguals, although this remains to be tested. Further, homesigns (gestures used by deaf individuals who lack conventional linguistic input) seem to be processed in the working memory of the homesigners who use them in much the same way, generally speaking, as words or lexicalized signs (Spaepen et al., 2013). Further investigation of working memory for homesigns could increase our understanding of the relation between working memory and language learning in the absence of a formal language system.

## Signal Degradation

**FIGURE 3:**
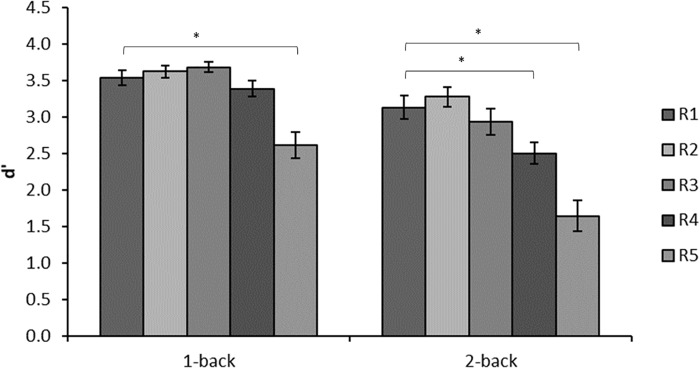
Mean d′ in each of the conditions of the n-back experiment. D′ is based on hits adjusted for false alarms in accordance with signal detection theory. Resolution decreases with increasing R. Reprinted from Rudner et al. (2015).

A common challenge to language understanding is the degradation of the incoming language signal that takes place in noisy conditions. This phenomenon has been widely researched in the speech modality (Rönnberg et al., 2013). However, it is not only acoustic noise that interferes with the speech signal, visual noise also interferes with speech perception (Cohen and Gordon-Salant, 2017) and the same applies to sign language perception (Pavel et al., 1987). In particular, reduced resolution introduced by signal compression in digital communication regularly used by sign language users may have a negative effect on communication quality (Agrafiotis et al., 2003). Indeed, visual noise in the form of reduced resolution negatively affects working memory for manual gestures and this effect interacts with working memory load such that poor signal quality has greater effect on n-back scores when load is higher (see **Table 1** and **Figure 3**, Rudner et al., 2015). A similar effect has been found for working memory for spoken words using alpha power as an index of working memory load (Obleser et al., 2012). This supports the notion that the effect of signal degradation on working memory is language modality general. However, the effect of signal degradation on working memory for gestures has only just started to be investigated, and more work is needed in this area.

## Implications for Working Memory Models

So far, this review has shown a range of working memory effects, some of which are specific to working memory for manual gestures and some of which are shared with working memory for spoken words. Most saliently, there is an effect of load on working memory for all types of manual gestures studied in this way (Spaepen et al., 2013; Rudner, 2015; Rudner et al., 2016b) and some evidence of an effect of signal degradation as well as an interaction between load and signal degradation (Rudner et al., 2015).

**FIGURE 4:**
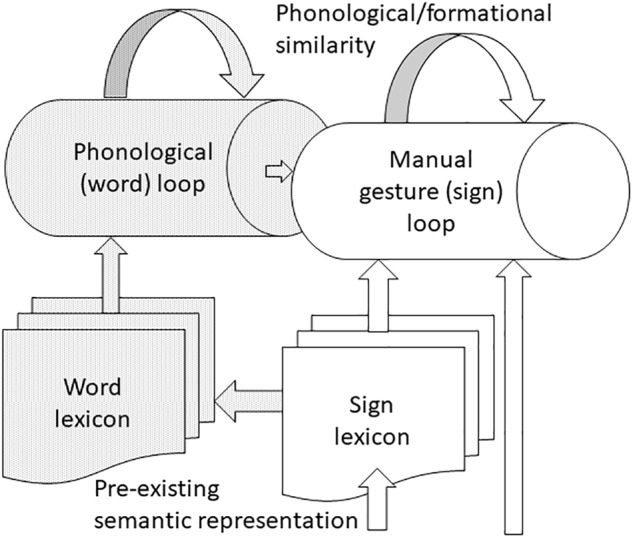
Working memory for linguistic and non-linguistic manual gestures. Manual gestures enter the corresponding loop either directly, if they are non-linguistic, or via the sign lexicon for individuals with pre-existing semantic representation. Bimodal bilinguals have the option of recoding signs as their word glosses and processing them via the word loop. Both loops are subject to negative effects of phonological/formational similarity, but while phonological familiarity aids processing in the word loop, this does not seem to be the case in the sign loop. Deafness leads to greater capacity of the sign loop, probably due to reliance on visual information and expert knowledge of sign language.

To this extent, effects on working memory are modality general. Although there is an effect of pre-existing semantic representation on working memory for manual gestures, the precise character of this effect is language modality specific and related to working memory load, see **Figure 4**. In particular, the effect of pre-existing semantic representation on working memory for manual gestures may only be apparent when load is high and the quality of representations maintained in working memory becomes particularly important (Rudner et al., 2016b). It is true that an effect of pre-existing semantic representation was shown for hearing signers with sign-based stimuli, but it is likely that familiar signs were recoded as words by these bimodal bilinguals for whom encoding and maintenance is likely to be more efficient in the oral rather than gestural modality (Hall and Bavelier, 2011; Rudner et al., 2016b). Further, there is a lack of strong evidence that pre-existing phonological representations are co-opted during working memory for manual gestures (Rudner et al., 2016b).

This pattern of findings is in line with flexible resource models of working memory (Ma et al., 2014). One such model is the Ease of Language Understanding model (ELU, Rönnberg, 2003; Rönnberg et al., 2008, 2013). This model explains the relationship between complex working memory and language understanding under challenging conditions. Originally (Rönnberg, 2003), ELU assumed a similar mechanism across the language modalities of sign and speech, but language modality specific aspects soon emerged (Rönnberg et al., 2008). The explicit cognitive processing that takes place when language understanding is challenged in various ways functions differently for sign and speech (Rudner and Rönnberg, 2008a,b; Rönnberg et al., 2008). On the other hand, the mechanisms underlying the implicit language understanding that takes place under optimal conditions seem to be similar across the language modalities of sign and speech (Rönnberg et al., 2000; MacSweeney et al., 2008a).

## Practical Implications – Cochlear Implantation

The differences between working memory for sign and speech that become apparent when language understanding is challenging have practical implications for a new generation of bimodal bilinguals who are also cochlear implant users. An increasingly common intervention for severe to profound deafness, both congenital and acquired, the cochlear implant (CI) transfers acoustic information collected via microphone at the scalp directly to the auditory nerve bypassing the defective inner ear. It allows individuals with deafness acquired post-lingually to preserve communication by restoring access to sound, albeit with a substantially degraded and distorted signal. Early implantation, from only a few months and no later than 7 years, allows many children with congenital deafness to acquire spoken language and cognitive skills, providing they have the right support (Tobey et al., 2011), and changes the course of cross-modal plasticity caused by deafness (Kral and Sharma, 2012; Glick and Sharma, 2017).

With the advent of cochlear implantation, many profoundly deaf children attend mainstream schools where there may be little opportunity to practice and develop sign language skills. In addition, only around 5% of deaf children who could benefit from sign language communication are born into deaf families where sign language is established and common place. This means, that many profoundly deaf children growing up today do not have the same access to sign language as their parents’ generation.

Children with CI perform at a lower level than their normal hearing peers on a wide range of cognitive tasks (Lyxell et al., 2008; van Wieringen and Wouters, 2015). These include short term memory measured using forward and backward digit span (Burkholder and Pisoni, 2003; Edwards et al., 2016) and visuospatial working memory (Beer et al., 2014), even though non-implanted deaf individuals have been shown to perform better than individuals with normal hearing on visuo-spatial working memory (Wilson et al., 1997; Geraci et al., 2008; Rudner et al., 2016a). It could be argued that standard administration of digit span (Wechsler Intelligence Scale) with oral presentation of stimuli would put individuals with the limited auditory access afforded by cochlear implants at a disadvantage. However, it seems that digit span discrepancies in children with cochlear implants are due to deficits in verbal rehearsal and serial scanning skills (Burkholder and Pisoni, 2003) rather than stimulus degradation as such (Carter et al., 2002; Burkholder-Juhasz et al., 2007). There is some evidence that children with cochlear implants in mainstream educational settings perform better cognitively than their peers in so-called total communication settings where speech is augmented with various kinds of visual cues although not typically sign language (Pisoni and Cleary, 2003; Tobey et al., 2011; Boons et al., 2012). However, because selection of educational setting is not random, care should be taken in interpreting this finding. Indeed, it has been shown that the speech development of deaf children with cochlear implants who have deaf signing parents and thus good access to sign language can be comparable to that of hearing peers (Davidson et al., 2014) although those with sign support in hearing families may not always do as well (Geers et al., 2017).

Early acquisition of language is vital for cognitive development (Mayberry et al., 2002) and may be the best predictor of successful language outcome for children born deaf (Campbell et al., 2014). Not only are sign languages fully ledged natural languages, they show similar developmental milestones to spoken languages and provide a good basis for their subsequent acquisition (Mayberry et al., 2002). Experience of sign language from infancy organizes the brain for language (Rönnberg et al., 2000; MacSweeney et al., 2008a; Campbell et al., 2014; MacSweeney and Cardin, 2015) and animal studies show that reorganization of auditory cortex for visual processing does not preclude subsequent auditory processing when cochlear implantation provides access to sound in the mature brain (Land et al., 2016). Although cochlear implantation is a revolution in the treatment of deafness, it provides only partial access to the richness of the speech signal, and in noisy situations it provides only limited assistance in segregating the signal of interest. Demonstrably, it provides a basis for language acquisition and cognitive development for many deaf children, but this basis is suboptimal. As Campbell et al. (2014) pointed out in their Frontiers review, there is little evidence to suggest that encouraging sign language development in deaf children is detrimental to speech development. If sign language can provide early and better quality cognitive representations leading to better ability to imitate gestures and maintain them in working memory its use should be stimulated.

Reading is a vital skill for everyone in the modern world but especially for deaf individuals for whom it can give access to information that may be less available through direct communication channels of sign and speech. Good language skills lay the foundation for good reading skills and this is true of both spoken and signed language (Holmer, 2016). A recent review by Mayer and Trezek (2017) shows that overall, studies of reading comprehension suggest that the majority of participants with cochlear implants achieved scores in the average range, although with a wide range of variability. The language skills of deaf native signing children are likely to be more firmly established for sign language than for a spoken second language acquired via cochlear implants. There is evidence that sign language skill predicts reading ability (Hermans et al., 2008; Holmer et al., 2016a), while the predictive strength of spoken language skills in deaf children is unreliable (Mayberry et al., 2010). Further, there is a link between reading ability and precision in imitating signs in deaf children (Holmer et al., 2016b). Recently, it has been shown that training the link between sign language and the written word may have a positive effect on word reading (Holmer et al., 2017). Thus, both spoken language skill and reading skill in deaf children are associated with firmly established first language skills.

## Future Directions

The investigation of working memory for manual gestures as an independent phenomenon rather than in comparison to working memory for words has only just begun and future directions of interest are many and various. I will outline some of the most salient.

This review reports evidence of an effect of semantic representation on working memory for manual gestures but no effect of phonological representation. The effect of semantic representation differed for deaf and hearing signers being apparent across different memory loads for hearing signers but only apparent for deaf signers at high memory load. Based on the emerging model of working memory for linguistic and non-linguistic manual gestures, see **Figure 4**, future work should investigate:

(1) the load limits of working memory for manual gestures in deaf signers and how they are influenced by pre-existing semantic representation.
(2) the influence of pre-existing semantic representation on working memory for manual gestures in hearing signers when sign-based representation is mandatory.
(3) the influence of pre-existing phonological representation when phonological representation is mandatory.
(4) the neural networks underpinning exploitation of pre-existing representation during working memory for manual gestures.

This review also reports effects of load and degradation on working memory for manual gestures similar to those found for words. Future work should investigate:

(5) the modality specificity of the neural networks underpinning effects of load and degradation and their interaction.

Little work has investigated the effect of age on working memory for manual gestures. Future work should investigate:

(6) how age plays into the phenomena listed above.

I have discussed how representation and maintenance of gesture may support language development in deaf children. Future work should investigate:

(7) imitation of, and memory for, manual gestures in deaf children as well as their correlation with academic development.

Other populations with disorders of language and cognition including but not limited to individuals with intellectual disabilities, apraxia, aphasia or psychiatric disorders such as schizophrenia may also benefit from using gesture as means of representation. Thus, future work should investigate:

(8) imitation of, and memory for, manual gestures in other clinical populations.

The ability to represent and maintain manual gestures in older adults at risk of post-lingual deafness has, to my knowledge, not yet been investigated. Future work should consider

(9) how age-related hearing loss plays into the above mentioned phenomena.

## Author Contributions

The article was devised and written by MR.

## Conflict of Interest Statement

The author declares that the research was conducted in the absence of any commercial or financial relationships that could be construed as a potential conflict of interest.
